# Idiopathic intracranial hypertension in males

**DOI:** 10.17712/nsj.2017.3.20170005

**Published:** 2017-07

**Authors:** Hissah K. Al Abdulsalam, Abdulrazag M. Ajlan

**Affiliations:** *From the College of Medicine (Al Abdulsalam), Department of Neurosurgery (Ajlan), King Saud University, Riyadh, Kingdom of Saudi Arabia, and from the Department of Neurosurgery (Ajlan), Stanford University, United States of America*

## Abstract

Idiopathic intracranial hypertension (IIH) is a neurological condition characterized by increased intracranial pressure without an underlying intracranial pathology. This condition is rarely encountered in men and it predominantly affects obese women of childbearing age. We present an interesting case of IIH in a male patient who presented with acute severe vision loss over 2 weeks and was successfully treated via surgery. Although IIH is less common in men than in women, men are more likely to develop vision loss, which is usually severe and less likely to respond to medical treatment. Therefore, surgical intervention might be considered early in the treatment of men with rapidly progressive visual loss. Further prospective studies are needed to evaluate the role of early surgical intervention in comparison to medical treatment in this group of patients.

Idiopathic intracranial hypertension (IIH), also known as pseudotumor cerebri (PC), is a neurological condition that is characterized by increased intracranial pressure (ICP) and normal cerebrospinal fluid (CSF) composition in the absence of an underlying intracranial pathology.^1^ This condition is rarely encountered in men and it predominantly affects obese women of childbearing age.^2^ Although IIH is less common in men than in women, men are more likely to develop severe vision loss;^3^ it has been suggested that men tend to have a more severe visual prognosis when compared to women.^2^,^4^ We therefore describe a case of IIH in a male patient who presented with acute severe vision loss. The patient was successfully treated with surgery and made a significant recovery.

## Case Report

A 21-year-old male presented with global headache for 4 weeks, as well as bilateral progressive vision loss for 2 weeks. He had no other neurological symptoms. Other than his presenting symptoms, his systemic evaluation was insignificant. He is otherwise healthy and he was not taking any medications. On examination, his body mass index was 38 kg/m^2^. He was alert, oriented to time and place, and his vitals were normal. On ophthalmic examination, he had decreased visual acuity to hand motion in the right eye and light perception in the left eye. His cranial nerves and extraocular motility were normal, with the exception of bilateral limitations on abduction. Fundus examination showed marked disc elevation with hyperemia, as well as hemorrhage of the optic discs in both eyes. Lumbar puncture was performed under fluoroscopy, which revealed clear CSF with an opening pressure of 55 cm H_2_O. Cerebrospinal fluid analysis revealed normal cell counts, as well as normal protein and glucose values. The radiological evaluation of the brain, including magnetic resonance imaging (MRI) and a computed tomography (CT) venogram, showed patent venous system, bilateral widening of the optic nerve sheath and a partially empty sella (**Figures A-D**). A diagnosis of IIH was established following Dandy’s modified criteria.^5^ The patient was subsequently started on medical treatment (acetazolamide, 2 g/day). There was no improvement of the patient’s visual deterioration in response to medical treatment over 2 days. Therefore, due to the rapidly progressive visual deterioration despite medical treatment, an emergency ventriculoperitoneal shunt (VPS) was inserted under neuronavigation (**Figures E and F**).

**Figure 1:**
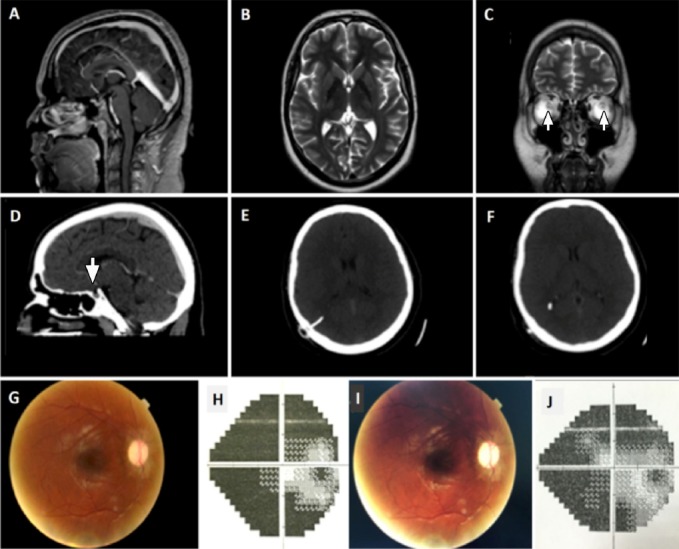
Imaging, fundoscopic pictures and visual field of the patient **A)** Sagittal T2-weighted MRI. **B)** Unremarkable axial T2-weighted MRI. **C)** Coronal T2-weighted MRI image demonstrates bilateral widening of the optic nerve sheath. **D)** CT venogram of the patient showing a partially empty sella. **E, F)** CT of the brain, 1 day postoperatively, demonstrating VP shunt insertion. **G)** Fundoscopic picture showing improved optic disc edema in the patient’s right eye (3 months postoperatively). **H)** Humphrey visual field test showing residual constriction of the patient’s peripheral vision in the right eye (3 months postoperatively). **I)** Fundoscopic picture of the patient’s right eye (7 months postoperatively). **J)** Humphrey visual field test showing some improvement of the patient’s peripheral vision in the right eye (7 months postoperatively).

The patient’s headache immediately improved postoperatively. His vision deterioration stabilized postoperatively. The patient was then discharged and was maintained on oral acetazolamide (1 g/day). He was subsequently followed in clinic 2 weeks postoperatively, where he reported no headaches and gradual improvement of his vision. At 3 months postoperatively, his visual acuity was 20/100 (right eye) and 20/200 (left eye), with some residual constriction of his peripheral vision on a visual field test (**Figure H**). Fundus examination at 3 months and 7 months postoperatively showed improved optic disc edema (**Figures G and I**). At 7 months postoperatively, his Humphrey visual field test showed some improvement of his peripheral vision (**Figure J**).

## Discussion

The IIH typically occurs in overweight women between the ages of 15-40 years; it rarely occurs in men.^2^ An epidemiological study conducted in the United States estimated that women are 8 times likely to be affected by IIH than men, with an annual incidence of 0.3/100,000 in men aged 15-44 years old, as compared to women of the same age (3.5/100,000).^6^ However, the incidence of IIH probably differs from region to region due to variations in the prevalence of obesity in each region. In a study preformed in a Middle Eastern country (Oman), the estimated annual incidence was 2.18/100,000 in the general population with women being 3 times more likely to be affected than men.^7^ The typical presentation of IIH includes signs and symptoms of increased ICP.^5^ Furthermore, IIH can uncommonly present with a fulminant presentation, which is defined as an acute presentation featuring severe vision loss that occurs over the course of several days.^8^

Although the clinical manifestations of IIH in men were reported to be similar to those in women, men were more likely to present with severe visual acuity and visual field defects when compared with women.^2^,^4^ Furthermore, Bruce^3^ and his colleagues reported that men tend to experience a shorter amount of time between the onset of the first symptom to the time of diagnosis when compared to women; however, this could be attributed to the more severe vision loss experienced by men at the time of presentation. Interestingly, it was reported that men are more likely than women to require surgical intervention for visual impairment.^2^ PubMed was searched using the following search terms: “Pseudotumor cerebri”, “Idiopathic intracranial hypertension”, “Men”, “Man”, “Male”, and “Males”. Relevant manuscripts were retrieved and the results were used to provide an overview of the extant literature. We found 47 males with IIH reported in the literature.^2^,^4^

A diagnosis of IIH is made with the aim of excluding any intracranial pathology. The modified Dandy diagnostic criteria were applied to our patient, as modern imaging techniques (such as MRI or CT) were used to exclude space-occupying lesions and ventricular enlargement. Additionally, MRI venography or CT venogram must be performed to exclude venous sinus thrombosis.^5^ An increased opening pressure without any CSF abnormalities is the typical diagnostic picture of IIH.^5^

The aim of treatment for patients with IIH is to preserve their vision and to reduce increased ICP.^1^ Weight loss can decrease the symptoms associated with IIH; however, with visual field deficits, weight loss alone is insufficient for decreasing ICP within the appropriate time frame.^1^ Of the 47 male patients who were reported in the literature, only one patient was treated with weight loss alone and showed no improvement.^2^

Patients without vision loss are usually treated with a carbonic anhydrase inhibitor, such as acetazolamide, as a first-line treatment to decrease increased ICP. Most patients typically respond to doses of 1–2 g, and higher doses of up to 4 g may be used, but some patients cannot tolerate the side effects.^1^ If the patient cannot tolerate acetazolamide, an alternative anhydrase inhibitor (such as methazolamide or furosemide) or other diuretics can be used. In our patient, described above, the maximum dose of acetazolamide given was 2 g per day over 2 days. However, due to the rapidly progressive visual deterioration of our patient, we decided to treat him surgically. Of the 47 patients examined herein, 24 of them were treated with acetazolamide without surgical intervention; of these, 17 presented with headache (64.7% of those patients improved with medical treatment) and 12 presented with visual symptoms (66.7% of those patients responded to medical treatment). However, in patients with acute visual field loss, or those who responded poorly to medical treatment, emergency surgical intervention should be considered. Surgery in the form of optic nerve sheath fenestration (ONSF) or shunting is indicated when patients have significant vision loss on presentation, which is refractory to maximum medical treatment.^9^ In patients who predominantly present with vision loss that is refractory to medical therapy, ONSF is usually preferred. On the other hand, for those patients who predominantly present with headache and who have failed to respond to medical therapy, CSF diversion is the preferred option.9 Of our 47 patients, 10 of them were treated surgically (7 with ONSF, 2 with lumbar–peritoneal shunts, and one who had both an ONSF/lumbar–peritoneal shunt); 60% of those 10 patients showed some improvement following surgical treatment.

It has been suggested that men tend to have more severe visual outcomes when compared to women.^2^,^4^ Interestingly, a recent randomized placebo-controlled trial of acetazolamide reported a significant association between male gender and medical treatment failure.^10^ The study was conducted to predict the risk factors that were associated with progressive vision loss in patients with IIH; these factors included low-sodium and weight-reduction diets. Patients in the trials were randomly allocated to receive either acetazolamide or placebo, and they were then followed for 6 months. Of all participants (147 females and 4 males), 7 patients (2 of which were men) met the criteria for treatment failure, with an odds ratio of 26.21 (95% confidence interval [CI]: 1.61-433.00; *p*=0.02) for males compared to females.^10^ Our report supports the role of early surgical intervention in men with severe vision loss. However, due to the limited reported cases, which were treated surgically, further studies are necessary to support early surgical intervention in this group of patients. Additionally, to our knowledge, the exact time between vision deterioration and the time at which vision can be aggressively preserved is not known.

In conclusion, although IIH is less common in men than in women, men are more likely to develop vision loss, which is typically severe and less likely to respond to medical treatment. Surgical intervention might be considered early in the treatment of men with rapidly progressive visual loss. Further prospective studies are needed to evaluate the role of early surgical intervention in comparison to medical treatment in this group of patients.
